# Comparative impact of polystyrene, rice bag-derived high-density polyethylene nanoparticles, and polystyrene–silver nanoparticle interactions in a 28-day in vivo study in male and female Wistar rats

**DOI:** 10.1038/s41598-026-35219-9

**Published:** 2026-01-19

**Authors:** Katarzyna Dziendzikowska, Malwina Czerwińska, Wojciech Grodzicki, Michał Oczkowski, Tomasz Królikowski, Joanna Gromadzka-Ostrowska, Sylwia Męczyńska-Wielgosz, Katarzyna Sikorska, Dariusz Kamola, Rafał Sapierzyński, Marcin Kruszewski

**Affiliations:** 1 Department of Dietetics, Institute of Human Nutrition Sciences, Warsaw University of Life Sciences, Nowoursynowska 159C, 02-776 Warsaw, Poland; 2 Centre for Radiobiology and Biological Dosimetry, Institute of Nuclear Chemistry and Technology, Dorodna 16, 03-195 Warsaw, Poland; 3 The Kielanowski Institute of Animal Physiology and Nutrition, Department of Genetic Engineering, Polish Academy of Sciences, Instytucka 3, 05-110 Jabłonna, Poland; 4 Department of Pathology and Veterinary Diagnostic, Institute of Veterinary Medicine, Warsaw University of Life Sciences, 02-776 Warsaw, Poland; 5 Department of Molecular Biology and Translational Research, Institute of Rural Health, Jaczewskiego 2, 20-090 Lublin, Poland

**Keywords:** Nanoplastics, Polystyrene nanoparticles, Polyethylene nanoparticles, Genotoxicity, Silver nanoparticles, Sex-specific toxicity, Biochemistry, Environmental sciences, Nanoscience and technology

## Abstract

Exposure to plastic nanoparticles (PNPs) has become a significant public health and environmental concern due to their pervasive presence and potential toxic effects. However, the long-term effects of different PNPs types, their interactions with other nanoparticles, and effects across sexes, remain poorly understood. This study aimed to evaluate sex-specific physiological, biochemical, and genotoxic effects of chronic exposure to polystyrene nanoparticles (PS-NPs), silver nanoparticles (AgNPs), high-density polyethylene nanoparticles (HDPE-NPs) isolated from food packaging, and a mixture of PS-NPs and AgNPs in male and female rats. Nanoparticles were administered daily for 28 days via oral gavage, after which selected systemic, metabolic, and genotoxic endpoints were assessed. Despite no overt systemic toxicity or major liver damage, we found changes in cholesterol levels, especially in females, and signs of DNA damage, suggesting potential genotoxicity. The combination of PS-NPs/AgNPs triggered liver stress responses, implying additive or synergistic effects. Importantly, females showed greater sensitivity in terms of lipid metabolism, whereas HDPE-NPs-treated male group reduced testicular weight. These findings underscore the necessity of including both sexes in nanoparticle toxicity studies and highlight the need for a better understanding of the health risks of nanoplastics and their interactions with other co-occurring contaminants under realistic exposure conditions.

## Introduction

In recent years, environmental pollution caused by plastic has increased dramatically. This issue affects mostly the aquatic environment, but humans are also exposed to nano- and micro-sized plastic particles, mostly through inhalation and ingestion of pollutants^1^. Plastic nanoparticles, which are invisible to the naked eye, are defined generally as a material with sizes ranging from 1 to 100 nm, sometimes from 1 to 1000 nm. These plastic particles, known as nanoplastic (PNP), have raised serious environmental and health concerns due to their widespread presence, environmental persistence, and possible interactions with organisms, including potential toxicity^2–4^. This PNP primarily originates from two sources: a breakdown of larger plastic debris, or intentional production by humans for use in personal care products or industrial processes, from which they are directly released to the environment^5^. Human exposure to PNP comes from various sources, including contaminated air, seafood tainted with plastic, beverage bottles, plastic food containers, and other food packaging material, such as plastic bags used for the distribution of food products, such as tea, rice, or groats^6^, but also intentionally added to cosmetics and medicines^1^. PNPs is present in surface and deep water, soil, and air, rendering its avoidance nearly impossible^7^. Plastic particles can also serve as carriers for other harmful substances, such as heavy metals or organic pollutants, affecting their bioavailability and toxicity^8^. Moreover, exposure to PNPs occurs alongside various other environmental pollutants and xenobiotics, including different types of nanomaterials^4^.

Due to their small size, PNP can penetrate biological barriers and enter organisms, potentially leading to negative effects at the cellular and molecular levels. This ability of PNP to infiltrate biological systems is particularly worrying because of the challenges associated with their detection and their potential biological activity^6^. The bioactivity and potential toxicity of PNPs depend on many factors, including their size, shape, chemical composition, and surface charge - all of which can affect their interactions with biological systems^9,10^.

The long-term health effects of continuous exposure to PNPs remain unknown, underscoring the critical need for comprehensive studies to understand their toxicological potential^7^. Furthermore, high-density polyethylene (HDPE) and polystyrene (PS) are among the most commonly used plastic materials, yet comparative toxicological data on these materials in nanoparticle form are scarce. This is especially true for environmentally relevant particles, such as HDPE-NPs, derived from consumer goods packages. Consequently, the present study was designed to deepen our understanding of the implications of PNPs exposure through in vivo research. This study aims to assess the biological impacts of polystyrene and rice bag-derived PNP on various health parameters in male and female rats. The parameters, evaluated after a 28-day exposure period, included body weight, hematological profiles, DNA integrity, and blood biochemistry. Moreover, having in mind a very likely scenario of simultaneous exposure to different nanomaterials, thereby amplifying their environmental risks and toxicological effects. Our study also investigated the combined exposure to polystyrene nanoparticles (PS-NPs) and silver nanoparticles (AgNPs). These two nanomaterials are among the most prevalent nanoparticles contaminating the environment and frequently coexist in aquatic and terrestrial systems due to the extensive use of silver-based products and the continuous degradation of plastic waste^11–14^. In water, AgNPs readily adsorb to the surface of PS-NPs, forming stable complexes. In this interaction driven by surface chemistry, PS-NPs act as a carrier that significantly influences the transformation, stability, and bioavailability of AgNPs, thereby modifying their environmental fate^11^. Moreover, the coexistence of these nanoparticles may enhance the dissolution of AgNPs, promote the formation of secondary particles, and alter their reactivity, contributing to increased toxicological risks^11,13,15^. Co-exposure to AgNPs and PS-NPs has been reported to enhance toxic effects in aquatic organisms, including increased silver accumulation and altered oxidative or hepatotoxic responses^16,17^. Therefore, our present study evaluates multiple toxicological endpoints, with particular emphasis on sex-related differences, to provide insight relevant to real-world exposure scenarios and inform risk assessment strategies in the field of nanotoxicology.

## Materials and methods

### Characterization and isolation of nanoparticles

#### Nanoparticles characterization

Spherical polystyrene nanoparticles (PS-NPs) with a nominal diameter of 20 ± 5 nm were obtained from NANOCS (New York, NY, USA) and were used here as a model plastic nanoparticle. The AgNPs used in this study were used and characterised in earlier studies conducted by our group^18–20^. The AgNPs, with a nominal diameter of approximately 20 ± 5 nm, were purchased from PlasmaChem (Berlin, Germany). All types of NPs investigated were thoroughly characterized in terms of size, shape, and dispersion quality. The physicochemical properties of AgNPs and the methods used to prepare the working suspensions have been comprehensively detailed in the referenced publications, and a concise overview is presented in Table 1. Electron microscopy images of identical AgNPs have been published previously^18,19^. To prepare the working suspension of AgNPs (5 mg/mL), 5 mg of silver nanopowder were suspended in 800 µL of distilled water and sonicated for 3 min, delivering a total ultrasound energy of 420 J/m³. Immediately thereafter, 100 µL of a 10× concentrated phosphate-buffered saline (PBS) solution and 100 µL of a 15% bovine serum albumin (BSA) solution were added to stabilize the dispersion. This preparation procedure was repeated before each administration to the animals to ensure reproducibility and uniformity of the exposure medium.

#### Isolation of high-density polyethylene nanoparticles from perforated films

High-density polyethylene plastic nanoparticles (HDPE-NPs) were extracted from perforated polymeric films, obtained from PLASTEXIM (Tychy, Poland), using a standardized thermal release and filtration procedure. PLASTEXIM is a supplier of HDPE film used for the packing of rice, groats, flakes, and other dry foods commercially available on the market in Poland. Initially, a 15-meter section of perforated film - equivalent to approximately 100 commercial storage bags intended for cooking of rice or groats - was cut into approximately 1 cm² fragments using stainless steel scissors. The resulting film fragments were transferred into a borosilicate glass vessel containing 2 L of deionized water and were heated on a magnetic stirrer for 1 h at 95–100 °C. After this extraction period, the film fragments were removed from the vessel, and the resultant aqueous suspension was concentrated by evaporation to a final volume of approximately 10 mL. The concentrated sample was then passed through a syringe filter featuring a 0.22 μm pore size, effectively isolating the suspended PNP and preventing bacterial contamination. The filtrate containing nanoparticles was subsequently collected and stored under appropriate conditions for further physicochemical and morphological characterization.

#### Characterization of nanoparticles

##### Morphological and size characterization of nanoparticles

The morphological features of the isolated HDPE-NPs and model polystyrene nanobeads were examined using high-resolution scanning electron microscopy (HR-SEM, Carl Zeiss “ULTRA plus,” Jena, Germany). Briefly, 100 µL of each aqueous sample was carefully deposited onto 12.5 mm diameter aluminum stubs with short pins. To ensure electrical conductivity and mitigate thermal damage during imaging, the samples were coated with a thin carbon layer using a vacuum evaporator (JEE-4X, JEOL, Tokyo, Japan). Micrographs were acquired at optimized beam parameters to precisely visualize particle size, shape, and surface topography.

Nanoparticle tracking analysis (NTA) was performed using a NanoSight LM20 system (NanoSight, Amesbury, UK) equipped with a 640 nm laser. For each measurement, 1 mL of particle suspension was injected into the instrument’s sample chamber at ambient temperature. The hydrodynamic size distribution was analyzed using NTA 2.0 Build 127 software, which recorded and assessed the Brownian motion of over 200 tracked particles *per* measurement. This approach provided information on both particle concentration and size distribution profiles, enhancing the understanding of the NPs population.

##### Zeta potential and polydispersity index (PDI) measurements via dynamic light scattering (DLS)

Zeta potential and polydispersity index measurements were carried out at 25 °C in a DTS 1067 capillary cell using a Zetasizer NanoZS instrument (Malvern, UK). Before measurement, the working solution was diluted at a ratio of 1:8 with deionized water. Each sample was measured in triplicate with 20 sub-runs to ensure reproducibility. Zeta potential value was calculated using the Smoluchowski approximation of the Henry equation (f(ka) = 1.5), which provided insights into the surface charge and colloidal stability of the particles. The polydispersity index (PDI) was determined from the autocorrelation function under default software parameters (filter factor: 50%, thresholds: 0.05 and 0.01), indicating the degree of heterogeneity in particle size distribution.

### Animals and in vivo experimental design

**Fig. 1:**
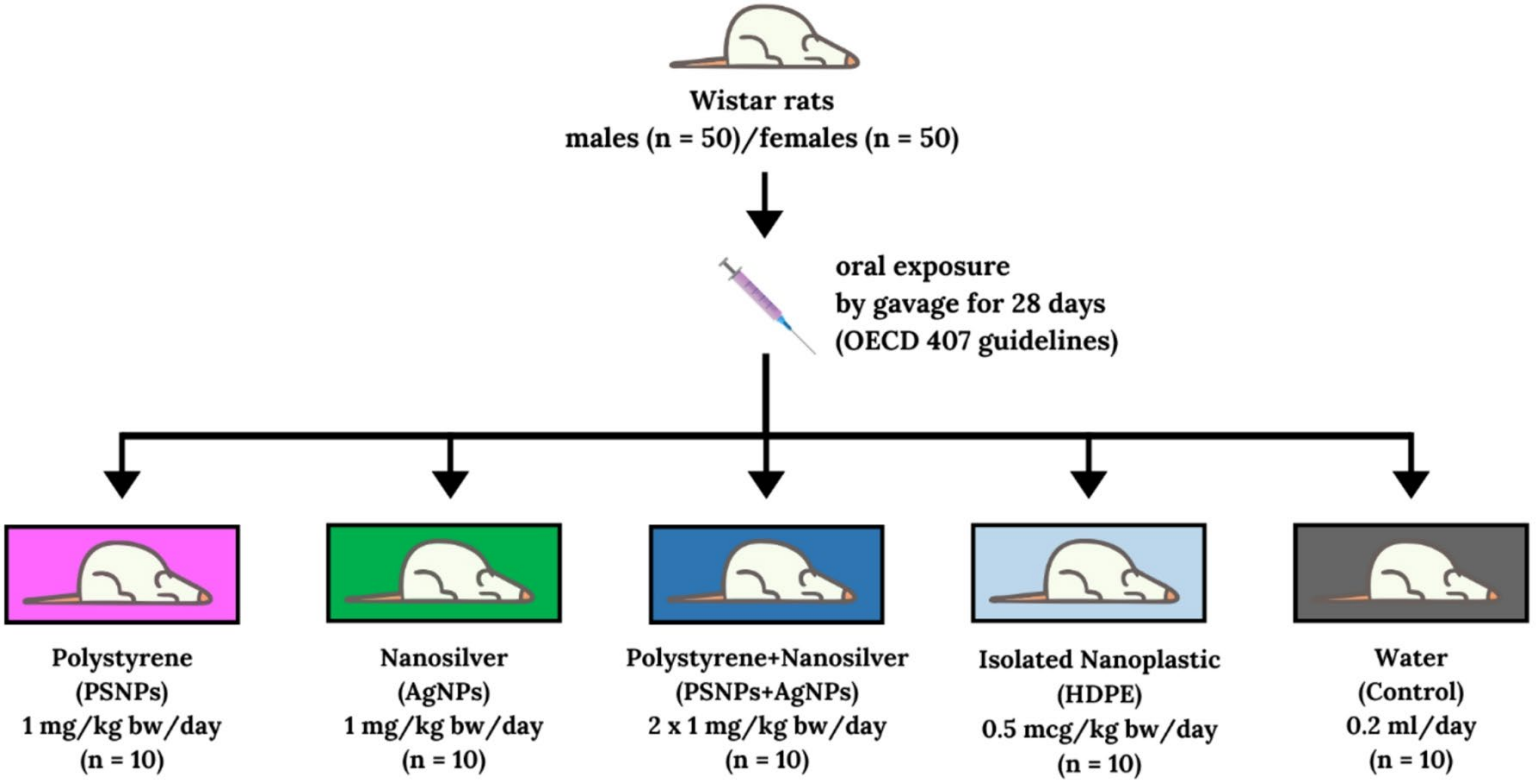
Scheme of the experimental design. Designed using elements by ©Canva via http://www.canva.com (accessed on 2-03-2025). The syringe icon represents the delivery of nanoparticle suspension via oral gavage.

The in vivo study was divided into two parts, one for each sex. The experiments were conducted on 7-week-old outbred Wistar Wu rats [Crl: WI(WU)], with an equal number of males (*n* = 50) and females (*n* = 50), purchased from Charles River Laboratories (Charles River, Sulzfeld, Germany). The rats were specific pathogen-free (SPF), with initial body weights of 275.72 ± 8.06 g for males and 180.96 ± 10.51 g for females. Outbred Wistar Wu rats [Crl: WI(WU)] are widely recognized as a versatile model in toxicological research, used to simulate human genetic variation and assess potential responses to environmental pollution. Animals of both sexes were kept in standard conditions featuring a 12-hour light/dark cycle, a stable temperature of 22°C, and 55% relative humidity. The rats were housed in polyurethane cages, two or three per cage, and had unrestricted access to water and food (Altromin 1324 maintenance diet, comprising 65% of energy from carbohydrates, 11% from fat, and 24% from protein, with a metabolizable energy content of 3.227 kcal/kg). Environmental enrichment in the rats’ cages was achieved through the use of glass balls and wooden round objects. Following a one-week acclimatization, rats were randomly allocated into one of the following experimental groups: (1) administered with 1 mg/kg body weight (bw) of commercial polystyrene nanoparticles (PS-NPs, *n* = 10); (2) administered with 1 mg/kg bw of silver nanoparticles (AgNPs, *n* = 10), which served as the positive control; (3) administered with a combination of both NPs (1 mg/kg bw PS-NPs + 1 mg/kg bw AgNPs, *n* = 10); (4) administered with 0.5 µg/kg bw of plastic nanoparticles extracted from consumer-grade rice bags made of high-density polyethylene (HDPE-NPs, *n* = 10); or administered with water (0.2 mL, *n* = 10), as the negative control. The NPs were administered orally *via* gavage (18ga x 75 mm) (Instech Laboratories, Inc. Plymouth, PA, USA) attached to a syringe, which enabled precise control of the administered volume and dose. Administration was performed once daily for 28 days following OECD 407 guidelines^21^. Animal body weight was recorded weekly throughout the experiment’s duration. All procedures were approved by The Second Local Committee for Ethics in Animal Research at the Warsaw University of Life Sciences (approval number WAW2/102/2021, 14 July 2021) and were conducted in accordance with the UE Directive (2010/63/UE), the Polish law regulation and with respect to the 3R (Replacement, Reduction and Refinement) rules. The key methodological elements emphasized in the ARRIVE 2.0 guidelines were implemented during the planning and conduct of the experiment. The number of animals in each experimental group was determined in accordance with the methodology proposed by Dell et al.^22^, using tools designed for estimating the sample size in in vivo experiments. The sample size (*n* = 8 per sex per group for biochemical, hematological, and genotoxic endpoints) was based on power calculations for a two-factor ANOVA design (sex × exposure type), supported by effect size estimates from our previous nanoparticle studies. Power analyses performed using the pwr2 package in R statistical software (www.rproject.org) indicated that this sample size provides adequate power (> 0.8) for detecting moderate to large effects and acceptable power for endpoints with lower expected variability. Two additional animals per sex were included for qualitative histological assessment. An illustration of the experimental design and group descriptions can be found in Fig. 1. Following the experiment, the animals were anesthetized by inhalation of Isoflurane (Aerrane Isoflurane USP, Baxter S.A, Lessines, Belgium) in an induction chamber saturated with Isoflurane vapor and subsequently euthanized by cardiac puncture under deep anesthesia. Throughout the experimental period, no symptoms indicating a disturbance in the well-being of the animals were observed; therefore, humane endpoints were not applied. To achieve hormonal and metabolic standardization of females, a natural method for synchronization of the estrous cycle was employed, specifically male odor-induced estrous cycle synchronization. This method involved housing of females in cages containing bedding from male cages throughout the entire experiment. After 28 days of the experiment, two males and two females from each group were deeply anesthetized with inhaled Isoflurane and perfused transcardially via the left ventricle, first with physiological saline and then with 4% paraformaldehyde solution. An Ismatec pump (ISM831C; Ismatec, Wertheim, Germany) was used for perfusion. Subsequently, the selected tissues were sampled and subjected to histological and immunohistochemical analyses. For males, testis weight was measured after necropsy as a non-specific marker for spermatogenesis and reproductive toxicity. For all molecular and biochemical analyses, each group comprised eight animals. Peripheral blood samples were collected in EDTA-coated tubes, and blood plasma was obtained by centrifugation at 2300× g for 10 min at 4 °C. The resulting plasma samples were subsequently stored at − 80 °C until needed for biochemical analyses. Haematological analysis was performed using freshly collected peripheral blood samples. The liver samples were preserved using 10% neutral buffered formalin, processed through paraffin embedding, sectioned at a thickness of 5 μm, and subsequently stained with hematoxylin and eosin (H&E) for evaluation by light microscopy.

### Estrous cycle determination

The phases of the estrous cycle were determined through vaginal smears collected immediately before necropsy, following terminal bleeding. According to OECD guidelines (No. 151, 2013), a cotton swab moistened with saline was inserted into the vagina to collect cells from the vaginal lumen and walls. The collected cells were then transferred into a clean glass slide. Vaginal smears were obtained at consistent time points between 8:00 AM and 12:00 AM, air-dried, and subsequently stained with 0.1% crystal violet for one minute, following the method described by McLean et al.^23^.

Microscopic analysis of the slides was conducted using a LEITZ Labovert FS inverted microscope equipped with 4×, 10×, 20×, and 32× objective lenses (Labovert FS Leitz, Wetzlar, Germany). Three distinct cell types were identified in vaginal smear samples: (1) nucleated epithelial cells, (2) cornified epithelial cells, and (3) leukocytes. The four stages of the estrous cycle (proestrus, estrus, metaestrus, and diestrus) were classified based on the presence, absence, or relative proportions of epithelial cells, cornified (keratinized) cells, and leukocytes, as described by Cora et al.^24^. Proestrus smears were characterized by moderate to high numbers of nucleated epithelial cells, while estrus smears consisted predominantly of cornified cells. Metestrus smears contained a large number of leukocytes alongside a smaller proportion of non-nucleated epithelial cells, whereas diestrus smears were primarily composed of leukocytes with varying numbers of epithelial and small cornified cells^24,25^.

### Haematological and biochemical analysis

Peripheral blood collected from the heart was divided into two portions: one as whole blood and the other for blood plasma. The whole-blood fraction underwent haematological evaluation using an Abacus Junior Vet analyzer (Diatron, Budapest, Hungary). This evaluation encompassed the quantification of total white blood cells (WBC), monocytes and eosinophils (MID), lymphocytes (LYM), granulocytes (GRA), red blood cells (RBC), and platelets (PLT). In addition, measurements were taken for mean corpuscular volume (MCV), red cell distribution width (RDW), mean corpuscular hemoglobin concentration (MCHC), mean corpuscular hemoglobin (MCH), hemoglobin concentration (HGB), and hematocrit (HCT). Plasma enzyme activities of alanine (ALT) and aspartate (AST) aminotransferases were measured using kinetic assays with commercially available kits (Sigma Aldrich, Saint Louis, MO, USA). In addition, plasma concentrations of cholesterol (CHO) and triglycerides (TG), were determined by colorimetric methods, employing commercially available kits (Sigma Aldrich, Saint Louis, MO, USA).

### Alkaline comet assay

The comet assay (single-cell gel electrophoresis) was conducted following a protocol described by Wojewódzka et al.^26^, modified to accommodate two gels per microscope slide. In brief, whole blood was sampled via cardiac puncture and collected into EDTA-coated tubes. A 10 µL of blood was mixed with 200 µL of 1.05% low melting point agarose (Type VII, Sigma–Aldrich, St. Louis, MO, USA), and 100 µL of the resulting suspension was layered onto each half of a microscope slide pre-coated with 0.5% regular agarose (Type I-A, Sigma–Aldrich, St. Louis, MO, USA). Coverslips were applied, and the slides were placed on ice to solidify the agarose. Once solidification was complete, the slides were immersed in a chilled lysis solution (2.5 M NaCl, 100 mM Na_2_EDTA, 10 mM Tris, and 1% Triton X-100, pH 10). After a 40-minute lysis, the slides were transferred to a horizontal electrophoresis unit containing fresh electrophoretic buffer (1 mM Na_2_EDTA and 300 mM NaOH) and left in this solution for 40 min to allow DNA unwinding. Electrophoresis was then run at 1.2 V/cm for 30 min at 4–6 °C. Upon completion, the slides were rinsed in 0.4 M Tris (pH 7.5) three times for 5 min each and stained with DAPI (1 µg/mL in PBS). Image capture and analysis were performed with the Comet Assay IV Image Analysis System (Perceptive Instruments, UK), evaluating 100 randomly selected comets per slide and quantifying DNA damage as the percentage of DNA in the comet tail.

A similar approach was employed for assessing DNA base damage, as described by Kruszewski et al.^27^, except that after lysis, slides were washed three times (5 min each) in buffer (40 mM Hepes, 0.1 M KCl, 0.5 mM EDTA, 0.2 mg/mL BSA, pH 8) at 4 °C. Subsequently, 60 µL of FPG enzyme solution (4.8 × 10^-2 U, New England Biolabs, UK) in the same buffer was applied to each slide, covered with a cover glass, and incubated at 37 °C in the dark for 30 min. Following incubation, DAPI staining (1 µg/mL in PBS) and comet analysis were carried out as described above.

### Statistical analysis

Experimental data were analysed using Statistica software v. 13.3 (StatSoft, Tulsa, OK, USA). Body mass gains were analysed by a three-way ANOVA with repeated measures (factors: exposure type, sex, and weeks with repeated measures on weeks), followed by Tukey’s post-hoc test for multiple comparisons. Haematological and biochemical parameters were assessed by two-way ANOVA with exposure type and sex as factors, followed by Dunnett’s post-hoc test (for comparisons with the corresponding control group) and Tukey’s post-hoc test (as indicated in figures and table legends). Statistical significance was set at *p* < 0.05. Normality of residuals was verified using the Shapiro–Wilk test, while homogeneity of variance was assessed using Brown–Forsythe’s test. Parameters that showed deviations from normality or heteroscedasticity were log-transformed before statistical analysis. All results are presented as mean ± standard error of the mean (SEM) of non-transformed values.

## Results

### Characterization of HDPE-NPs, PS-NPs, and a mixture of PS-NPs/AgNPs

**Fig. 2:**
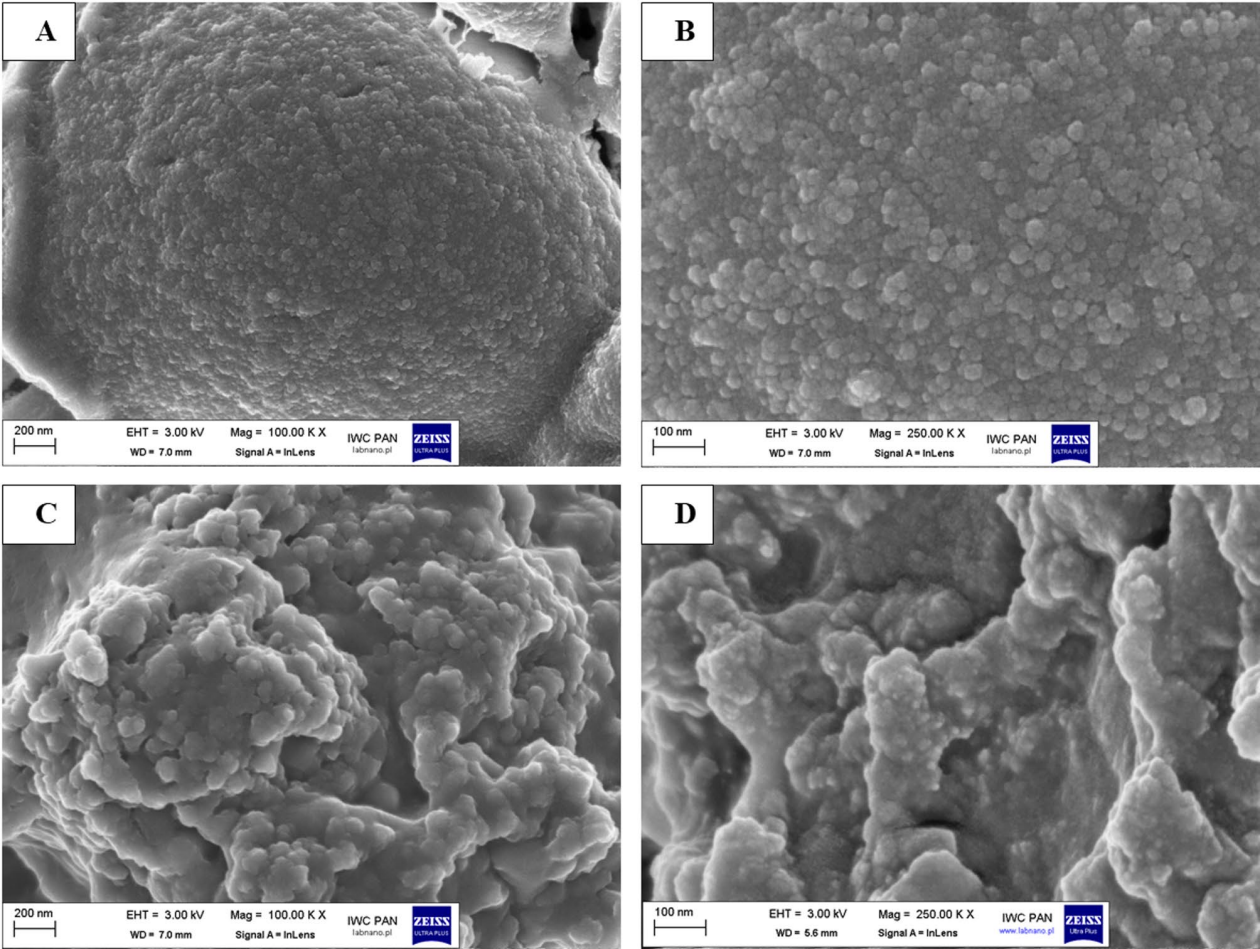
Scanning electron microscope (SEM) images of plastic nanoparticles. (**A**) SEM image of polystyrene nanoparticles (PS-NP) (20 nm), 100.00 KX, (**B**) SEM image of PS-NP (20 nm), 250.00 KX, (**C**) SEM image of HDPE-NPs, 100.00 KX, (**D**) SEM image of HDPE-NPs, 250.00 KX.

The morphology, shape, and dimensional characteristics of the investigated PS-NPs were evaluated using scanning electron microscopy (SEM). Representative images of the commercial polystyrene beads are shown in Fig. 2A and B. These beads tended to form substantial, irregularly shaped aggregates with a morphology reminiscent of petal-like structures. At higher magnification (Fig. 1B and 250.00 KX), individual NPs approximately 20 nm in diameter were discerned in proximity to these larger agglomerates, in agreement with the manufacturer’s specifications (NANOCS, New York, NY, USA).

SEM examination of HDPE-NPs (Fig. 2C and D) revealed their tendency to cluster into irregular aggregates with rough, uneven surfaces. Moreover, these aggregates exhibited micro-scale voids and jagged edges, indicative of structural heterogeneity. The aggregated particle sizes within these samples were generally between 100 and 200 nm, suggesting a broader size distribution, as compared to the near-monodisperse polystyrene references.

The agglomerates were subjected to a 10-minute ultrasonic treatment before further measurements to achieve a more uniform dispersion. Table 1 summarizes the physico-chemical parameters of nanoparticles and their mixtures used in this study. The PS-NPs exhibited an average hydrodynamic diameter of 62.4 ± 31.6 nm. In contrast, the HDPE-NPs were larger, with an average diameter of 217.0 ± 24.1 nm. Polydispersity index indicated relatively high homogeneity of AgNPs and PSNPs suspension, whereas HDPE-NPs suspension was less homogenic.

**Table 1:** Characteristics of nanoparticles (measured in deionized water)^18–20^.

	AgNPs	Polystyrene NPs	Mixture (PS-NPs/Ag-NPs)	HDPE-NPs
Nominal size of particles [nm]	20 ± 5	20 ± 5	–	–
Hydrodynamic diameter [nm]	197.4 ± 2.7	62.4 ± 3.1	290.1 ± 34.1	217.0 ± 24.1
Polydispersity index	0.295 ± 4.2	0.231 ± 0.24	0.701 ± 0.63	0.580 ± 0.44
Zeta potential [mV]	-33.6 ± 3.5	-40.02 ± 2.41	-31.23 ± 1.09	-33.92 ± 1.02
BET surface area [m^2^/g]	2.2419	ND	ND	ND
Micropore volume [cm^3^/g]	0.0076	ND	ND	ND
Adsorption average pore width (nm)	13.6698	ND	ND	ND
Desorption average pore width (nm)	23.8934	ND	ND	ND

Data expressed as mean ± SD (*n* = 3). ND – not determined, due to the limited amount of material.

### Animal condition and body weight

In this study, we assessed the general toxicity and systemic effects of PNP particles derived from polystyrene and consumer-grade rice bags, along with the impact of co-exposure to PS-NPs and AgNPs. The experiment included five groups: PS-NPs, AgNPs, a combination of PS-NPs and AgNPs (PS-NPs/AgNPs), HDPE-NPs extracted from consumer-grade rice bags, and a control group. The study followed OECD 407^21^ guidelines for toxicity assessment in rodents. Body weight gain was monitored weekly, and daily animal health condition observations were conducted to evaluate systemic effects associated with exposure to relatively low doses of NPs.

**Fig. 3:**
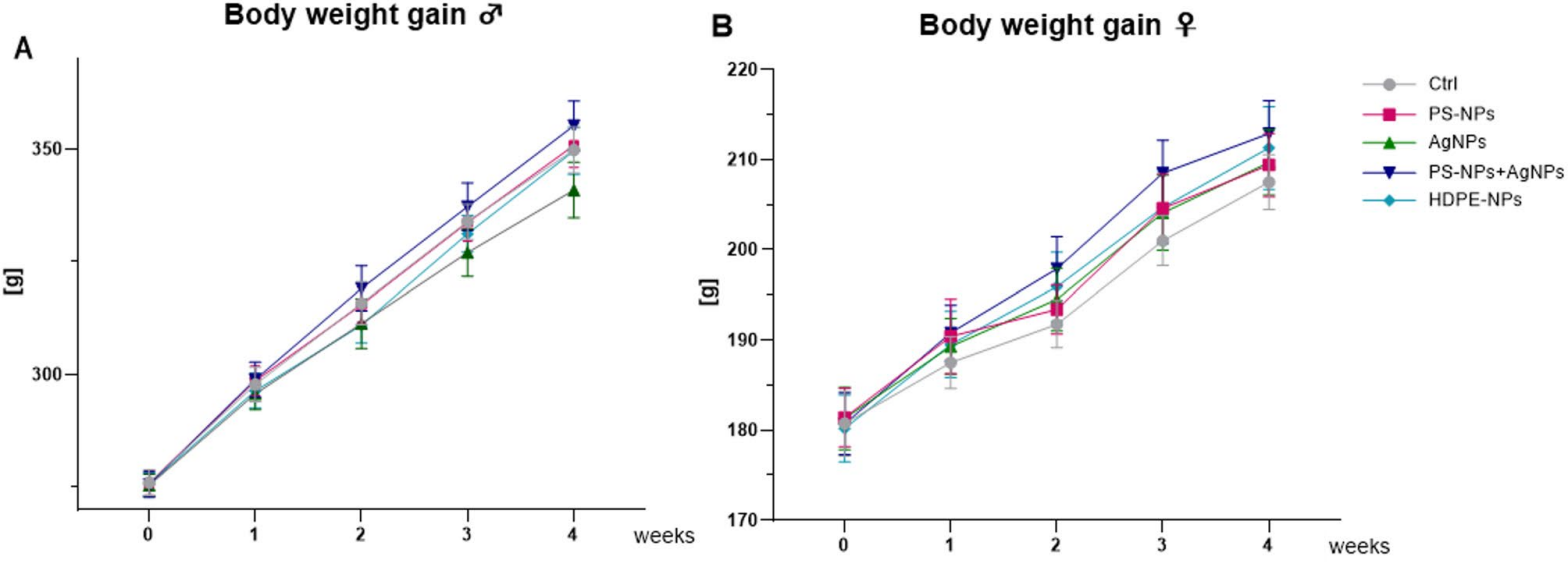
Body weight gain during 4 weeks of experiments in male (**A**) and female (**B**) [mean ± SE]. Repeated-measures ANOVA: effect of time *p <* 0.001, effect of sex *p <* 0.001; time × exposure interaction *p <* 0.001, time × sex interaction *p* < 0.001.

Throughout the experiment, no clinical signs of systemic toxicity were observed, and the overall condition of animals in both the experimental and control groups remained comparable. Post-mortem macroscopic examination did not reveal any abnormalities in adipose tissue distribution or overt pathological alterations in major organs, including the liver, kidneys, or spleen. Specifically, no evidence of steatosis, inflammatory infiltrates, and hyperaemia was detected. All animals exhibited normal growth and developmental patterns (Table 2). Analysis of initial and final body weight, as well as weekly % of body weight gain, revealed that these growth-related parameters were significantly affected by the sex of the animals, however, without any influence of nanoparticle exposure. Since weekly % body weight gain represents an average rate normalized across the four weeks, the effect of time is mathematically removed from this variable. A more detailed temporal analysis of weekly body weight dynamics is presented below (Fig. 3).

The weight of the testis was evaluated as an initial marker of reproductive toxicity. The ANOVA analysis revealed a significant effect of nanoparticle exposure on the testis weight, and Tukey’s post hoc test showed a significantly lower testicular weight in male rats exposed to HDPE-NPs compared to controls (Table 2).

**Table 2:** Animal body weight, body weight gain and testis weight.

	Sex of animals	Subgroup					ANOVA
		Control	PS-NPs	AgNPs	PS-NPs + Ag-NPs	HDPE-NPs	
Initial body weight [g]	Male	276.0 ± 2.8	275.9 ± 2.6	275.4 ± 2.4	275.6 ± 2.8	275.7 ± 2.5	E: NS S: *p* = 0.0001 E x S: NS
	Female	179.6 ± 3.4	181.4 ± 3.2	181.3 ± 3.5	180.7 ± 3.4	180.2 ± 3.7	
Final body weight [g]	Male	349.0 ± 5.3	350.8 ± 4.9	341.9 ± 6.5	355.2 ± 5.5	349.6 ± 5.2	E: NS S: *p* = 0.0001 E x S: NS
	Female	207.5 ± 3.1	209.4 ± 3.5	209.7 ± 3.6	212.9 ± 3.7	211.3 ± 4.6	
Body weight gain [%/week]	Male	6.05 ± 0.24	6.19 ± 0.20	5.54 ± 0.34	6.55 ± 0.21	6.11 ± 0.23	E: NS S: *p* = 0.0001 E x S: NS
	Female	3.54 ± 0.34	3.73 ± 0.36	3.73 ± 0.30	4.23 ± 0.45	4.08 ± 0.34	
Testes weight [g]	Male	3.43 ± 0.06	3.43 ± 0.06	3.31 ± 0.06	3.46 ± 0.05	3.23 ± 0.04 *	E: *p* = 0.0198

The data are presented as mean ± SE, *n* = 10.

* Statistically significant difference from those of the control group according to Dunnett post-hoc test (*p* < 0.05).

ANOVA factors: E – NPs exposure; S – sex; E x S – interaction of both factors

A repeated-measures ANOVA was conducted to evaluate body weight development over the course of the 28-day exposure period. Statistical analysis confirmed a significant effect of time, indicating a progressive increase in body weight across all groups. Sex was also identified as a determining factor of body weight gain, with males consistently heavier than females. In addition, significant interactions were observed between time and exposure, as well as time and sex, indicating that body weight trajectories differed depending on both treatment and sex (Fig. 3). Tukey’s post hoc test revealed that male rats exhibited significantly higher body weight compared to age-matched females at all time points (*p <* 0.05 for all comparisons). Specifically, in male rats, body weight increased steadily from week 0 to week 4 in all exposure groups (*p <* 0.05). In contrast, in female rats, significant weight gain was observed only during the first and third week of exposure in all experimental groups (*p <* 0.05 for all comparisons), no significant changes were detected during the second and fourth weeks.

### Estrous cycle

Analysis of immediately post-mortem collected vaginal smears revealed that 26 females (65%) were in proestrus. It is important to note that the estrous cycle in female rats is relatively short (4–5 days). Therefore, the transition from proestrus to estrus was still classified as proestrus, as described by Cora et al.^24^. Typical estrus was observed in 4 females (10%), while both diestrus and metaestrus were identified in 4 females (10%) and 6 females (15%), respectively (Table 3).

Distribution of estrous cycle stages across the different experimental groups is presented in Table 3. The greatest variability in estrous stages was observed in the control group. The lowest variation was found in the group that received PS-NPs, indicating a moderate degree of success in the applied natural synchronization method.

**Table 3:** Classification of the estrous cycle stages in Wistar rat females.

Experimental group	Estrous cycle stages			
	Proestrus	Estrus	Metaestrus	Diestrus
Polistyrene nanoparticles (PS-NPs)	7	1	0	0
Silver nanoparticles (AgNPs)	7	0	0	1
PS-NPs/AgNPs	5	1	2	0
High-density polyethylene nanoparticles isolated from consumer-available rice bags (HDPE-NPs)	5	0	2	1
Control	2	0	4	2

The number of females in a particular phase of the estrous cycle is indicated by the numbers in the columns.

### Nanoplastic effect on blood haematological parameters and blood biochemistry - physiological status of animals

Haematological analyses were performed to assess the impact of NPs exposure and sex in rats. The results for all analyzed peripheral blood parameters are presented in Table 4. The WBC counts were significantly influenced by both NPs exposure and sex, with a significant interaction between these two factors. Overall, males exhibited higher WBC counts than females, reflecting typical sex-related differences. In females, exposure to AgNPs led to a significant reduction in WBC counts, in comparison with the control and PS-NPs groups, no exposure-related changes were observed in males. These findings suggest sex-specific variability in how circulating leukocyte levels respond to nanoparticles exposure.

The PLT was also significantly influenced by the type of administered NPs and sex, with no significant interaction. Males exhibited significantly higher PLT values. In females, no significant differences were observed between exposure groups. In males, increased PLT values were recorded in the PS-NPs and AgNPs groups compared to the PS-NPs/AgNPs group. However, these differences were not confirmed by Tukey’s post-hoc test.

**Table 4:** Haematological values (mean ± SE).

Feature	Sex of animals	Subgroup					ANOVA
		Control	PS-NPs	AgNPs	PS-NPs/Ag-NPs	HDPE-NPs	
WBC [10^9^/L]	Male	6.18 ± 0.37	5.92 ± 0.25	6.28 ± 0.09 ^a^	5.44 ± 0.23	5.57 ± 0.20	E: *p* = 0.0049 S: *p* = 0.0002 E x S: *p* = 0.0109
	Female	6.03 ± 0.31	5.74 ± 0.33 ^b^	4.27 ± 0.30 *^,a, b^	4.94 ± 0.25	4.91 ± 0.31	
PLT [10^9^/L]	Male	550.57 ± 18.31	578.83 ± 26.74	564.50 ± 20.91	498.85 ± 20.78	525.13 ± 12.61	E: *p* = 0.0309 S: *p* = 0.0056 E x S: NS
	Female	497.88 ± 14.81	534.25 ± 15.06	514.13 ± 15.95	507.00 ± 17.55	502.50 ± 14.49	
RBC [10^12^/L]	Male	8.49 ± 0.06	8.68 ± 0.06	8.40 ± 0.21	8.67 ± 0,13	8.36 ± 0.09	E: NS S: *p* = 0.0001 E x S: NS
	Female	8.37 ± 0.08	8.33 ± 0.08	8.05 ± 0.11	8.17 ± 0,10	8.23 ± 0.07	
HGB [g/dL]	Male	14.71 ± 0.20	14.90 ± 0.15	14.72 ± 0.26	14.96 ± 0.15	14.41 ± 0.14	E: NS S: NS E x S: NS
	Female	14.94 ± 0.12	15.04 ± 0.13	14.48 ± 0.17	14.84 ± 0.16	14.98 ± 0.13	
HTC [%]	Male	47.19 ± 0.49	48.10 ± 0.31	47.24 ± 0.94	48.40 ± 0.59	46.43 ± 0.49	E: NS S: NS E x S: NS
	Female	48.13 ± 0.44	47.86 ± 0.49	46.23 ± 0.59	46.84 ± 0.75	47.68 ± 0.42	
MCV [fl]	Male	55.57 ± 0.30 ^a^	55.29 ± 0. 29 ^b^	55.33 ± 0.33 ^c^	55.75 ± 0.25 ^d^	55.50 ± 0.27 ^e^	E: NS S: *p* = 0.0001 E x S: NS
	Female	57.63 ± 0.18 ^a^	57.63 ± 0.18 ^b^	57.50 ± 0.19 ^c^	57.50 ± 0.19 ^d^	57.75 ± 0.16 ^e^	
MCHC [g/dL]	Male	31.16 ± 0.24	31.36 ± 0.16	31.40 ± 0.23	31.06 ± 0.13	31.08 ± 0.16	E: NS S: *p* = 0.0350 E x S: NS
	Female	31.23 ± 0.10	31.39 ± 0.17	31.33 ± 0.14	31.54 ± 0.18	31.71 ± 0.13	
MCH [pg]	Male	17.33 ± 0.15 ^a^	17.24 ± 0.10 ^b^	17.24 ± 0.17 ^c^	17.33 ± 0.15 ^d^	17.26 ± 0.09 ^e^	E: NS S: *p* = 0.0001 E x S: NS
	Female	17.97 ± 0.03 ^a^	18.06 ± 0.11 ^b^	17.98 ± 0.12 ^c^	18.18 ± 0.10 ^d^	18.20 ± 0.13 ^e^	

WBC – white blood cells; PLT –platelet; RBC – red blood cells; HGB – hemoglobin; HCT – hematocrits; MCV – mean corpuscular volume; MCHC – mean concentration of haemoglobin in erythrocytes; MCH – mean content of haemoglobin in a single erythrocyte.

The data are presented as mean ± SE, *n* = 8. * indicates a significant difference versus the control group (Dunnett’s post hoc test, *p* < 0.05); Superscript letters (a–e) indicate significant differences between exposure groups (Tukey’s post hoc test, *p* < 0.05) including comparisons between nanoparticle treatments within the same sex, and between males and females within the same treatment group; identical letters mean groups that differ significantly from the same comparison group. ANOVA factors: E – NPs exposure; S – sex; ExS – interaction of both factors.

Erythrocyte counts (RBC) were significantly affected only by sex, with higher values observed in males. This reflects expected physiological dimorphism and was not related to NPs exposure. The most pronounced sex differences were noted between the male and female PS-NPs/AgNPs groups and between AgNPs-exposed females and PS-NPs/ AgNPs-exposed males. Similarly, red cell indices also demonstrated sex-related variation. Mean corpuscular volume (MCV) and mean corpuscular haemoglobin (MCH) were both significantly higher in females, although no significant effects of exposure or interaction were observed. No significant differences in MCV or MCH were observed between exposure groups within either sex. Similarly, mean corpuscular haemoglobin concentration (MCHC) was higher in females, without significant variation across exposure groups or interaction effects. No significant differences between exposure groups were observed within either sex. Haemoglobin concentration (HGB) and haematocrit (HCT) also did not differ significantly neither between males and females, nor among groups exposed to different NPs, further supporting the absence of exposure-related effects on red blood cell physiology.

**Fig. 4:**
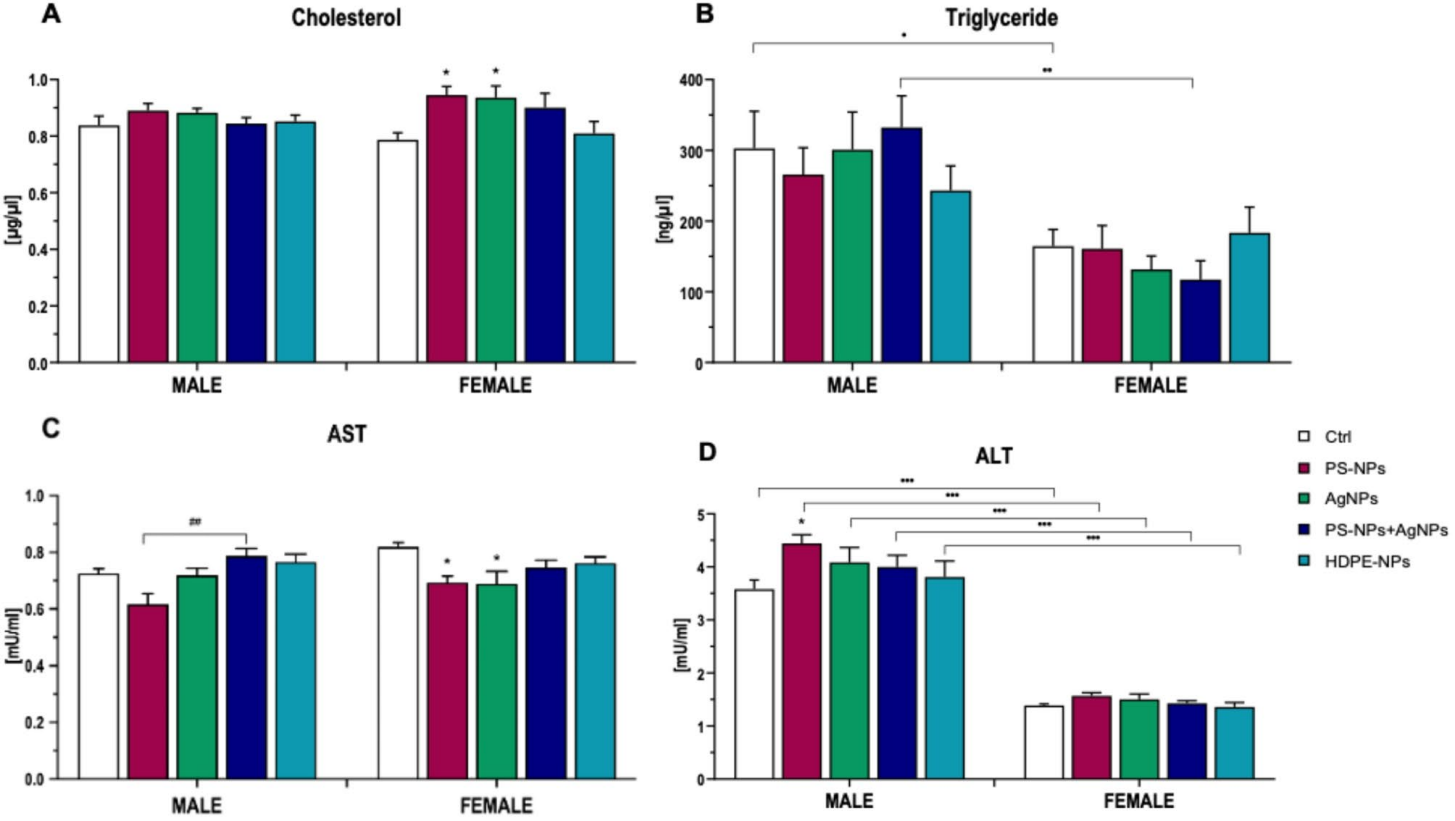
Plasma cholesterol (**A**), triglyceride (**B**), and plasma activity of aspartate (AST) and alanine (ALT) aminotransferase. Data are presented as mean ± SE. ANOVA results: Cholesterol: E: *p* < 0.005; S: NS; E x S: NS; Triglycerides: E: NS; S: *p* < 0.0001; E x S: NS; AST: E: *p* < 0.0001; S: NS; E x S: NS; ALT: E: *p* < 0.05; S: *p* < 0.0001; E x S: NS. ANOVA factors: E – NPs exposure; S – sex; E x S – interaction of both factors. ^*^ Statistically significant difference from the control group according to Dunnett post-hoc test (^*^ *p <* 0.05). ^●^ one-way ANOVA statistically significant difference from the corresponding exposure group of different sex according to Tukey post-hoc test (^●^ *p <* 0.05, ^●●^ *p <* 0.01, ^●^^●^^●^ *p <* 0.001). ^#^ one-way ANOVA statistically significant difference between exposure groups in the same sex according to Tukey post-hoc test (^##^ *p <* 0.01).

Plasma cholesterol (CHO) and triglyceride (TG) levels, along with the activities of liver enzymes, such as alanine aminotransferase (ALT) and aspartate aminotransferase (AST), are widely used as biomarkers for evaluating liver function and systemic toxicity. Alterations in these parameters can indicate hepatic injury, metabolic disturbances, or broader toxicological effects on the organism. In the present study, a significant effect of exposure was observed for CHO, indicating that NPs can modulate lipid metabolism. No significant differences were observed in males, whereas in female rats, plasma CHO level was significantly elevated in both the PS-NPs and AgNPs groups, as compared to the control group (Fig. 4A). This may suggest that females may be more sensitive to NPs-induced disturbances in cholesterol homeostasis. In contrast, TG concentration was strongly sex-dependent, with males displaying significantly higher TG concentration than females, despite the exposure. No exposure-related differences occurred within either sex, indicating that TG metabolism was not affected under the tested conditions (Fig. 4B). AST enzyme activity was significantly affected by NPs exposure, though not by sex or interaction. In female rats, AST activity was significantly reduced in the PS-NPs and AgNPs groups relative to control animals. In contrast, in male rats, plasma AST activity was significantly elevated in the PS-NPs/AgNPs group, as compared to the PS-NPs group (Fig. 4C). These findings may indicate sex-specific hepatic responses to different nanoparticle types or combinations. Regarding ALT activity, statistical analysis demonstrated a significant effect related to the NPs exposure and sex of the animals. Across all exposure groups, males consistently exhibited higher ALT activity than females. Within males, ALT activity was significantly elevated in the PS-NPs group, as compared to the control, indicating a mild hepatocellular response to PS-NPs exposure, consistent with a possible hepatocellular response. No significant difference between exposure groups was observed in females (Fig. 4D).

### Nanoplastics effects on liver histology

**Fig. 5:**
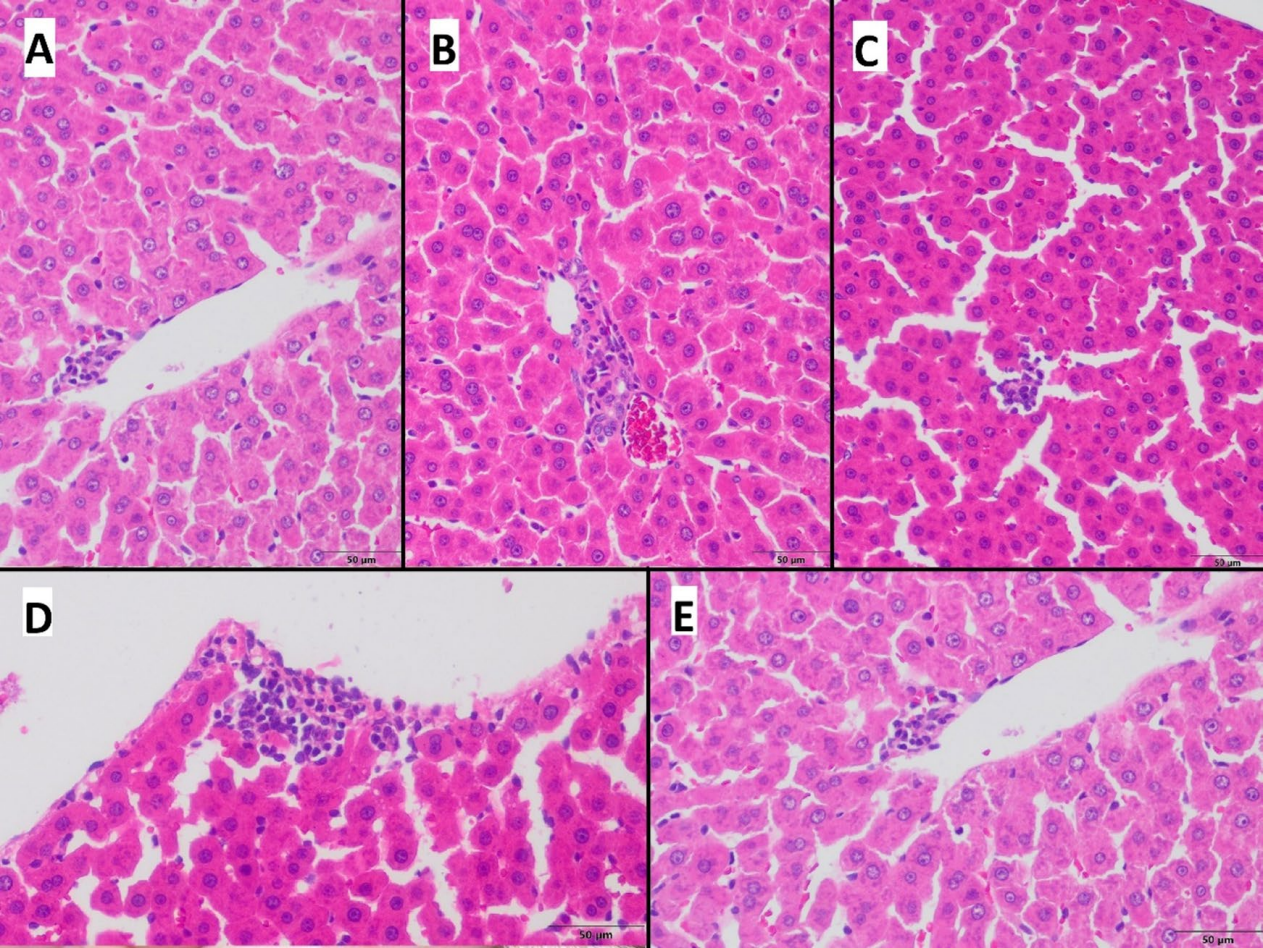
Representative examples of samples collected from livers of animals from groups: (**A**) PS-NPs, (**B**) AgNPs, (**C**) PS-NPs + AgNPs, (**D**) HDPE-NPs, and (**E**) control, presenting small foci of hematopoiesis within liver parenchyma, mostly perivascularly. Hematoxylin-eosin staining, magnification 200x.

Histological analysis of liver tissue from all experimental groups revealed a comparable microscopic morphology. Analyses demonstrated small foci of extramedullary hematopoiesis dispersed throughout the hepatic parenchyma (Fig. 5). Additionally, mild anisokaryosis of hepatocyte nuclei was observed, characterized by slight variability in nuclear size (Fig. 5). Notably, the extent and severity of these histopathological alterations were minimal and similar across all experimental groups (Fig. 5). No significant differences were detected among the groups, indicating that oral administration of different types of NPs under the experimental conditions did not induce considerable changes in the liver microstructure.

### Nanoplastics effects on DNA damage in the blood cells

**Fig. 6:**
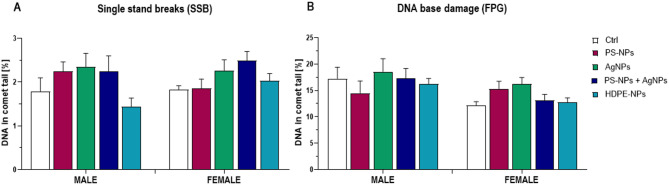
Single-strand breaks (SSB) (**A**) and DNA base damage are recognized by formamide-pyrimidine glycosylase (FPG) (**B**) in blood cells. The data are presented as mean ± SE. ANOVA results: SSB E *p* < 0.05; S: NS; E: x S: NS; FPG E: NS; S: *p* < 0.01; E x S: NS. ANOVA factors: E – NPs exposure; S – sex; E x S – interaction of both factors.

Statistical analysis revealed that single-strand breaks (SSBs) in blood nucleated cells of male rats varied significantly depending on the exposure factor, with a lower SSB level observed in the HDPE-NPs and control groups (Fig. 6A). Formamidopyrimidine DNA glycosylase (FPG)-sensitive sites, indicating oxidative DNA base damage, were sex-dependent, with higher values in male rats. No significant changes were observed as a result of exposure to different types of NPs (Fig. 6B). Although the ANOVA indicated a significant overall effect, no pairwise differences reached significance in the post-hoc tests.

## Discussion

The widespread occurrence and persistence of micro- and nanoplastic (PNP) in the environment, along with its confirmed presence in food packaging materials, have raised serious concerns about its potential impact on human health^28–30^. Recent studies have shown that PNP contamination can originate not only from environmental sources, but also from direct release of particles during the use of plastic food contact materials, such as plastic teabags^31,32^, infant feeding bottles^33^, and a water bottle^34^. Consequently, unintentional oral exposure appears inevitable, as evidenced by the detection of PNP in human faeces^35^, blood^36^, and even placental tissue^37^.

Although in vivo studies have investigated nanoplastic toxicity, research integrating direct comparisons between different nanoplastic types—including environmentally derived HDPE-NPs—and assessing sex-specific responses within a single controlled in vivo model remains limited. Therefore, the present study addresses this gap by assessing the effects of 28-day oral exposure to two types of PNP, PS-NPs and HDPE-NPs derived from consumer packaging, in a rat model. In addition to exposure to single NPs types, we also investigated the consequences of co-exposure to PS-NPs and AgNPs, mimicking a more environmentally relevant, multi-contaminant scenario. A broad spectrum of endpoints was evaluated, including body weight changes, haematological profiles, DNA integrity, blood biochemistry, and liver histology. Importantly, the study design included both sexes, enabling the detection of sex-dependent responses, an aspect often neglected in toxicological testing, despite its relevance for human risk assessment. Our findings contribute novel in vivo data on NPs toxicity and highlight the need for further investigation of the effects of NPs mixtures and sex-specific susceptibility, both critical for regulatory toxicology and health risk evaluation.

NPs dosing in this study was selected to reflect environmentally relevant exposure scenarios, allowing for toxicologically relevant comparison and, where applicable, to reflect environmentally possible exposure levels. PS-NPs and AgNPs were used at a dose of 1 mg/kg bw, in line with concentrations often applied in nanotoxicological in vivo studies. For AgNPs, this dose was chosen based on documented adverse effects in mammalian models, including studies conducted by our team and other authors^38,39^. In the co-exposure group (PS-NPs + AgNPs), the same dosing regimen (1 mg/kg bw of PS-NPs and 1 mg/kg bw of AgNPs) was applied to allow for direct assessment of potential additive or synergistic interactions and to maintain consistency in data interpretation across treatment groups.

In contrast, the HDPE-NPs were administered a substantially lower dose (0.5 µg/kg bw), selected to mimic an environmentally realistic high-end exposure scenario. Estimates of human intake of micro- and nanoplastics vary widely, with values ranging from 0.008 to 0.583 µg/kg bw per day, depending on exposure routes and food categories as reported by Mohamed Nor et al.^40^ Domenech and Marcos^41^ further estimated that humans may ingest up to 80 million particles *per* day. Based on standard human body weight assumption of 70 kg and applying density values from commercially available NPs, this corresponds to approximately 0.5 µg/kg bw *per* day. To ensure translational relevance, human-to-animal dose conversion as proposed by Nair and Jacob^42^ was applied to ensure the selected doses were as representative and translationally meaningful as possible. However, a substantial difference in administered doses (environmentally relevant for HDPE-NPs and toxicologically relevant for PS-NPs and AgNPs) does not allow for a direct comparison of toxicological potency between these nanoparticle types. In the present study, the PS-NPs, AgNPs, and PS-NPs/AgNPs groups were included to enable direct comparisons at equivalent doses, whereas the aim regarding HDPE-NPs was not to compare their toxicity with these materials but to determine whether HDPE-NPs, administered at environmentally realistic levels, induce detectable biological effects.

To ensure accurate interpretation of biological effects, a comprehensive physicochemical characterization of the tested nanoparticles was performed. Hydrodynamic diameters determined by nanoparticle tracking analysis (NTA) were 62.4 ± 3.1 nm for PS-NPs, 217.0 ± 24.1 nm for HDPE-NPs, and 290.1 ± 34.1 nm for the Ag-NPs/PS-NPs mixture. In all cases, the measured diameters were significantly larger than those observed by scanning electron microscopy (SEM). This difference arises from the distinct measurement principles of the two techniques. SEM provides the dry core size of individual particles under vacuum, while NTA measures particles in suspension, recording their Brownian motion and calculating the hydrodynamic diameter, which includes not only the solid core but also the surrounding solvation layer and any surface-bound molecules (nanoparticle corona).

Surface charge measurements conducted using Dynamic Light Scattering (DLS) revealed that all nanoparticles exhibited strongly negative zeta potentials: −40.02 ± 2.41 mV for PS-NPs, − 34.98 ± 1.98 mV for HDPE-NPs, and − 32.23 ± 3.92 mV for Ag-NPs/PS-NPs mixture. These values suggest high colloidal stability, as zeta potential values beyond ± 30 mV are generally considered favourable for maintaining stable nanoparticle dispersions, as they provide sufficient electrostatic repulsion to prevent aggregation^43,44^. Furthermore, the polydispersity index (PDI) values revealed significant differences in size distribution. The AgNPs and PS-NPs exhibited a relatively monodisperse system (PDI: 0.295 and 0.207, respectively), whereas isolated HDPE-NPs (PDI: 0.773) and Ag-NPs/PS-NPs mixture (PDI: 0.692) displayed a broader and more variable particle size distribution, which may influence their biological interactions.

The biological effects of NPs are closely related to their physicochemical characteristics, including size, surface charge, and dispersion stability, which affect gastrointestinal absorption and systemic distribution. In the present study, oral exposure to PS-NPs, AgNPs, HDPE-NPs, or a PS-NPs/AgNPs mixture for 28 days did not result in overt systemic toxicity or clinical signs of distress in rats. Although, toxicity of micro- and nanoparticles was previously observed^45–47^, the present study revealed that exposure of rats to different types of PNP, did not change animals’ overall health condition. In contrast to our results, some research has demonstrated that chronic PS-NPs exposure may impair metabolic function and nutrient absorption, leading to reduced body weight gain in offspring and juvenile mice^48,49^. These effects have been linked to gut microbiota alterations, inflammatory response, and increased oxidative stress. In line, subchronic oral exposure to AgNPs and PNP has been shown to disrupt gut microbiota composition and intestinal barrier integrity, contributing to systemic effects^50,51^.

A significant reduction in testicular weight was observed in male rats exposed to HDPE-NPs, a finding that, to our knowledge, has not been previously reported for this nanoparticle type. While most toxicological data on PNPs have focused on gastrointestinal, hepatic, or systemic effects, the current result suggests that HDPE-NPs may also affect the male reproductive system. Although no comparable studies are available for HDPE-NPs specifically, the observed effect aligns with previous studies indicating that AgNPs may adversely affect male reproductive organs through mechanisms involving oxidative stress, inflammation, or endocrine disruption^52,53^. Additionally, Yu et al.^54^ have demonstrated that prolonged oral exposure to leachates from boiled-water-treated plastic materials, including HDPE, can disrupt spermatogenesis and lipid metabolism, providing molecular-level evidence of plastic-associated reproductive toxicity. These findings underscore the potential reproductive risks associated with exposure to PNPs, necessitating further mechanistic exploration.

Our results indicate that the majority of haematological and biochemical blood parameters of rats exposed to PS-NPs, AgNPs, or HDPE-NPs remain within established reference values^55^ and showed no significant alterations resulting from exposure. These observations are consistent with previous studies reporting minimal effects of AgNPs exposure on blood parameters in animal models^56^. The observed sex-dependent differences in erythrocyte-related parameters (RBC, MCV, MCH) and platelet counts are likely attributable to inherent physiological factors, such as differences in hormonal regulation and iron metabolism between males and females^57^. The significant interaction between AgNPs and sex in modulating leukocyte counts supports earlier research suggesting sex-dependent inflammatory responses following exposure to AgNPs^58^.

Histopathological analysis confirmed that oral administration of PS-NPs, AgNPs or HDPE-NPs. did not significantly alter liver microstructure, indicating low hepatic toxicity of these nanoparticles at the tested doses. However, the variations observed in plasma CHO, TG, AST, and ALT levels indicate modest, sex-dependent shifts in metabolic and liver-related biochemical markers, although their biological relevance remains uncertain. In male rats, AST activity was significantly increased in the group exposed to the mixture of PS-NPs and AgNPs, as compared to animals receiving PS-NPs alone, indicating that combined exposure may result in additive effects on liver-related biochemical markers. This finding suggests that simultaneous exposure to both particle types may intensify hepatocellular stress beyond the level induced by PS-NPs alone. Recent research demonstrated that PS-NPs could markedly affect the transformation, stability, and toxicity of AgNPs in aquatic systems. Their co-occurrence may enhance AgNP dissolution, promote formation of secondary particles, and modify their environmental fate and bioavailability, thereby increasing toxicological potential^11,13,15^. These findings may help explain the enhanced AST response in our combined exposure group, although further mechanistic studies are needed to confirm this interaction in vivo. Elevated plasma cholesterol level observed in females exposed to PS-NPs and AgNPs may reflect subtle, exposure-related shifts in lipid metabolism, although the underlying processes remain unclear. Such metabolic disturbances could be mediated by nanoparticle-induced oxidative stress and inflammatory responses, which are among the primary mechanisms proposed to underlie nanoparticle toxicity. Nanoparticles have been shown to interfere with lipid metabolism through mechanisms involving oxidative stress and inflammation. For instance, PS-NPs can accumulate in the liver, where they induce oxidative damage, promote pro-inflammatory responses, and impair lipid metabolism, resulting in hepatic steatosis and fibrosis observed in both mammalian and aquatic models^50,59^. Similarly, AgNPs increased oxidative stress markers and inflammatory cytokines, together with alteration of plasma lipid profiles and inducement of hepatocellular injury in rodent models^60–63^. Mechanistic studies indicated that these effects were primarily mediated by the generation of reactive oxygen species and activation of inflammatory signaling cascades, which could disrupt lipid synthesis, transport, and storage^64,65^. The observed elevation in plasma cholesterol levels in females may reflect sex-specific regulation of lipid metabolism, which is strongly influenced by estrogens and differs from males in terms of lipid storage, distribution, and enzymatic control^66^.

Statistical analysis revealed that nanoparticle exposure significantly affected the induction of single-strand DNA breaks (SSBs) in blood nucleated cells (ANOVA, *p* < 0.05), with generally higher values observed in animals exposed to PS-NPs, AgNPs, and their combination, as compared to the HDPE-NPs-exposed or control groups. Although the post-hoc Tukey test did not confirm statistical significance of differences between individual exposure groups, the overall pattern suggested a potential genotoxic effect of PS-NPs and AgNPs, probably associated with increased oxidative stress. Similar nanoparticle-induced oxidative DNA base damage has been previously reported and linked to oxidative mechanisms involving the generation of reactive oxygen species and subsequent impairment of DNA repair pathways^67,68^. However, the absence of nanoparticle-type-dependent changes in FPG-sensitive sites may suggest that oxidative base damage depends strongly on inherent biological factors, such as sex-specific metabolic processes, rather than on the chemical nature of the nanoparticles themselves. Research shows that females generally exhibit greater resistance to oxidative stress-induced cell damage, which is attributed to both hormonal effects, such as estrogen enhancing antioxidant defence, and intrinsic cellular differences, including superior mitochondrial function and higher expression of stress response genes in female cells compared to males^69^.

In our study, to reduce hormonal and metabolic variability among female subjects, estrous cycle synchronization was performed using continuous exposure to male-derived olfactory cues by housing females with bedding previously used by males. This approach is supported by evidence that male scent can induce estrus or accelerate transitions between estrous stages in female rodents. Wölfl et al.^70^ demonstrated that exposure to male odor reliably induces estrus and advances cycle progression in wild female mice. However, their findings also underscore that olfactory-induced estrus does not produce full or lasting synchronization of estrous stages across individuals; rather, it triggers or accelerates specific cycle transitions without aligning all females to the same stage. In our present study, a high proportion of females in the experimental group, particularly among those exposed to single nanoparticles, were classified as being in proestrus. As expected for natural synchronization methods, the approach was only partially successful, with variability remaining across experimental groups, particularly in the control and PS-NPs/AgNPs mixture groups. Importantly, nanoparticle exposure did not appear to disrupt the regularity of estrous cycles in female rats, and the distribution of cycle stages did not correspond with treatment-related patterns in physiological, biochemical, or genotoxic endpoints. Although synchronization in the control group was somewhat less consistent, no corresponding increase in physiological variability was observed in the endpoints measured. Nevertheless, potential fluctuations in hormone-sensitive parameters, such as plasma lipid levels, hepatic enzyme activities (ALT, AST), and platelet counts, as well as, to a lesser extent, red blood cell indices (e.g., RBC, HGB, MCV), due to incomplete synchronization should be taken into account when interpreting toxicological outcomes and in the design of future studies. It should also be noted that incomplete synchronization typically increases inter-individual variability in hormone-sensitive endpoints, which can reduce statistical power and make subtle effects more difficult to detect. The fact that clear treatment-related differences were observed despite this inherent variability suggests that the detected effects represent robust biological responses rather than artifacts of hormonal alignment.

Interestingly, although most physiological and biochemical endpoints were unaffected by HDPE-NPs administered at an environmentally relevant dose, a significant reduction in testicular weight was observed in male rats. Notably, this effect was not observed in animals exposed to PS-NPs, AgNPs, or their mixture, despite these nanoparticles being administered at higher doses. The magnitude of the HDPE-NPs effect is therefore striking in the context of the very low administered dose. However, because the dosing regimens were intentionally designed to address different questions — environmental relevance for HDPE-NPs versus toxicological benchmarking for PS-NPs and AgNPs — no definitive conclusions can be drawn regarding their relative toxicological potency. The reduction in testicular weight cannot be interpreted as evidence of greater toxicity of HDPE-NPs, but may indicate that HDPE-derived nanoparticles elicit measurable biological effects, even at very low exposure levels. Future studies employing multiple dose levels will be necessary to clarify whether this response reflects higher potency per unit dose or arises from differences in physicochemical properties, bioavailability, or biological interactions. Importantly, polyethylene- and polystyrene-based nanoplastics differ not only in polymer type but also in their physicochemical characteristics, degradation behaviour, surface properties, and potential for biological uptake. These material-specific features may substantially influence biodistribution, cellular interactions, and downstream biological responses^71^. Dedicated studies are therefore needed to determine how polymer identity and particle properties contribute to the distinct biological effects observed in this and other studies.

Furthermore, sex-dependent differences in susceptibility to NPs-induced toxicity were observed in our study. Those results are consistent with evidence suggesting distinct physiological responses between males and females to environmental contaminants^58^. Further investigation into the exact mechanisms underlying nanoparticle interactions with metabolic regulation pathways is warranted. The sex-specific differences observed in ALT activity further suggest endogenous biological factors, such as hormonal influences or differential metabolic capacities, playing crucial roles in the hepatotoxic responses observed.

## Conclusion

In conclusion, chronic oral exposure to PS-NPs, AgNPs, or HDPE-NPs did not induce overt systemic toxicity or histopathological alterations; however, changes in cholesterol levels, particularly in females, together with indications of DNA damage suggest subtle metabolic disturbances and potential genotoxic effects.  Our findings emphasize the importance of incorporating both sexes in toxicological assessments, as the response to nanoparticle exposure differed markedly between male and female rats. Notably, female rats exhibited higher susceptibility, particularly demonstrated by imbalanced lipid metabolism. These sex-dependent variations underscore the necessity of evaluating both sexes for an accurate and comprehensive risk assessment of nanoparticle exposure. Notably, HDPE-NPs exposure led to a significant reduction in testicular weight, highlighting potential reproductive risks associated with certain types of nanoplastics. Further mechanistic investigations into HDPE-related reproductive toxicity are warranted. Additionally, our analysis of vaginal smears indicated high effectiveness of natural estrous cycle synchronization, with nanoparticle exposure seemingly not significantly disrupting the regularity of estrous cycles in female rats.

## Data Availability

The datasets used and/or analysed during the current study are available from the corresponding author on request.
